# Ghrelin antagonist overrides the mRNA expression of NPY in hypothalamus in feed restricted ewes

**DOI:** 10.1371/journal.pone.0238465

**Published:** 2020-09-09

**Authors:** Ana C. Carranza Martin, Anthony J. Parker, Cecilia C. Furnus, Alejandro Enrique Relling

**Affiliations:** 1 Department of Animal Sciences, The Ohio State University, Wooster, OH, United States of America; 2 IGEVET—Instituto de Genética Veterinaria Prof. Fernando N. Dulout (UNLP-CONICET), Facultad de Ciencias Veterinarias, Universidad Nacional de La Plata, La Plata, Buenos Aires, Argentina; Universidade Federal de Viçosa, BRAZIL

## Abstract

A negative energy balance (NEB) is detrimental to reproduction in animals. A suggested link between NEB and reproductive failure is the gastrointestinal hormone ghrelin, because of the association between ghrelin and the hypothalamo-pituitary-gonadal axis. The [D-Lys3]-Growth Hormone Releasing Peptide-6 ([D-Lys3]-GHRP-6) is a ghrelin antagonist that acts on ghrelin receptors (GHS-R1). The objective of this study was to evaluate the effect of [D-Lys3]-GHRP-6 on reproduction variables in feed restricted ewes. Two experiments were conducted. Experiment I was conducted for 30 days; and Experiment II for 13 days. In both experiments the ewes (n = 18) were randomly assigned to: Control (CO): fed to meet maintenance requirements; Feed restriction (FR): 80% of maintenance restriction; or Ghrelin antagonist (GA): feed restricted and daily subcutaneous of 7.5μg/kg of [D-Lys3]-GHRP-6. Plasma was collected to measure hormones and metabolite concentration. In Experiment II, the hypothalamus and ovaries were collected on day 13. In both Experiments, sheep allocated to the FR and GA treatments decreased their body weight compared with sheep in the CO group (P < 0.06); progesterone however, did not differ between treatments (P > 0.10). Experiment I: Plasma ghrelin concentration was greater (P < 0.01) in FR and GA compared with CO ewes. Plasma non-esterified fatty acids concentration was greater (P < 0.01) in GA and FR than CO. Experiment II: Kisspeptin1-Receptor (Kiss1-R) mRNA expression was greater in FR (P < 0.01) and tended to be greater in GA (P = 0.10) compared with CO ewes. The neuro peptide-Y (NPY) mRNA expression was greater (P = 0.03) in FR than CO; and tended to be greater (P = 0.06) compared with GA ewes. Growth hormone releasing hormone (GhRH) mRNA expression was greater in GA (P = 0.04) and tended to be greater in FR (P = 0.07) compared with CO ewes. Feed restriction increased GhRH, NPY, and Kiss-R mRNA expression in hypothalamus without affecting reproductive variables.Ghrelin antagonist may prevent an increase inNPY expression in ewes.

## Introduction

Ruminants affected by a negative energy balance (NEB) are subjected to metabolic change [1]. The metabolic change includes the regulation of complex signaling pathways that result in nutrient partitioning, and ultimately can impair the estrus cycle in cattle [1]. Plasma ghrelin concentration increases in sheep [2] and humans [3] during the pre-prandial period. In addition, plasma ghrelin concentration increases during feed restriction compared with rams given ad-libitum intake [4].Ghrelin, is an endogenous ligand for the growth hormone secretagogue receptor (GHS-R1a) [5]. In the brain, the binding of ghrelin to GHS-R1a stimulates growth hormone (GH) secretion [5], up-regulates growth hormone release hormone (GHRH) [6], and increases food intake regulating energy balance [7]. Ghrelin also plays a role in the hypothalamo-piuitary-gonadal axis. Ghrelin inhibits the secretion of gonadotropin release hormone (GnRH) in the hypothalamus of rats [8]. Additionally, ghrelin decreases plasma luteal hormone (LH) concentration in sheep [9]. The release of GnRH by the hypothalamus stimulates LH synthesis and secretion from the hypophysis. When both hormones, GnRH and LH, are affected there is a dysfunction on the reproductive cycle [10]. As a consequence, fertility and reproductive behavior of the animals are compromised. Another possible mechanism of how ghrelin regulates reproduction is by suppressing kisspeptin (Kiss1) secretion. Kisspeptin is a hypothalamic neuropeptide that stimulates GnRH release [11].

Ghrelin has been associated with an increase in mRNA concentration of orexigenic neuropeptides like neuropeptide Y (NPY) and agouti-related protein (AgRP) [4]; both neuropeptides increased feed intake and energy expenditure [12]. The NPY and AgRP neuropeptides not only regulate feed intake, but also, regulate the reproductive hypothalamus-piuitary-gonadal axis [13]. For example, the infusion of an NPY agonist decreased the release of gonadotropin in sheep [14].

The GHS-R1a receptor was immuno-localized in granulosa cells from follicles at all developmental stages and in luteal cells of corpus luteum (CL) in ewe ovaries [15]. The greatest expression of GHS-R1a in ovaries is during the CL development and lesser expression is during CL regressing phase [15]. In pigs, an increased in plasmaghrelin concentration has a negative effect on plasma progesterone concentration [16]. The effect of ghrelin on progesterone synthesized by the CL has not been reported in ewes. The direct effect of ghrelin in the CL could be one of the mechanisms by which NEB affects reproduction.

The [D-Lys3]-Growth Hormone Releasing Peptide-6 ([D-Lys(3)] -GHRP-6) is a ghrelin antagonist. I*n-vivo* and *in-vitro* experiments determine that [D-Lys3]-GHRP-6 binds to GHS-R1a [17, 18]. Some of the traits negatively affected by ghrelin were antagonized by [D-Lys 3]—GHRP-6, such as decreasing male sexual behavior, LH gene expression [19], and feed intake in rats [20].

We hypothesized that food restriction increases plasma ghrelin concentration. Furthermore, we hypothesized that ghrelin, by activating its receptor, compromises the reproductive cyclicity. This is due to changes on the mRNA of energy balance neuropeptides, reproductive neuropeptides, hormones, and their receptors in the hypothalamus; and modifying the mRNA of stereological enzymes in ovaries. As a consequence, ewes in NEB will have a low plasma progesterone concentration. However, those effects are suppressed by blocking ghrelin activity with [D-Lys3]-GHRP-6. The objective of the present study was to evaluate the effect [D-Lys3]-GHRP-6 in reproduction variables in feed restricted ewes.

## Materials and method

All animal procedures were approved by the Agricultural Animal Care and Use Committee of The Ohio State University (IACUC # 2016A00000089). The Experiments were conducted in the sheep research center of the Ohio Agricultural Research and Development Center. To test our hypothesis, we carried out two experiments.

### Animals, treatments, and sampling for Experiment I

This Experiment was performed to evaluate one complete estrus cycle. During this period plasma progesterone concentration and ovarian activity were evaluated.

During the breeding season eighteen ewes (80.1 ± 1.56 kg and 2.5–3 in a five scale of body condition score; [21]) were allotted in pairs to pens and synchronized with prostaglandin (estrumate, PG600, Merck, Washington, IA, USA) at days 1 and 9 (1.7μg/kg, IM) as described by Gottfredson [22]. Day 1 was considered the first day of the experiment. Each pen was randomly assigned to one of the following three treatments: (i) Control (CO): ewes fed a diet that provided the maintenance nutrient requirements for a ewe and given a daily subcutaneous (SC) saline infusion (0.1mL/kg body weight,BW); (ii) Feed restriction (FR): ewes were fed a diet restricted to 80% of maintenance requirements and given a daily subcutaneous (SC) saline infusion (0.1mL/kg BW); (iii) Ghrelin antagonist (GA): the same restricted diet as the FR and given a daily subcutaneous (SC) ghrelin antagonist infusion (7.5μg/Kg BW of [D-Lys3]-GHRP-6 diluted into 0.1ml/kg BW of saline solution; Phoenix Pharmaceuticals, INC. Burlingame, CA, USA). Antagonist dose used was based previous studies [23, 24].

The experiment was conducted over 30 days. During this period ewes were infused daily before feeding (between 0800 to 0900). For the CO group the feed offered was a mixed diet containing 2kg of corn silage, 0.225 kg of soy hull and 0.09 kg of distillers grains with solubles, totaling of 2.3 kg animal feed/day. Ewes in the FR and GA groups were given 80% of the maintenance diet (1.76 kg/animal/day, 20% restriction). There was no feed refusal during the length of the study. The chemical composition and nutrient analysis of the diet is described in Table 1.

**Table 1:** Ingredient and nutrient composition of the diet fed to the ewes in Experiments I and II.

Item	Composition (%)
Corn silage^1^	86
Soybean meal^2^	3.51
Soy hulls^3^	8.77
Mineral and vitamin supplement	1.72
**Nutrient, % of DM**	
NDF_a_	45.59
EE	1.75
CP	10.8
ASH	4.52
NE m ^4^/, Mcal/kg	1.6

^1^Contained 36.14% DM, 4.87% CP, 1.6% EE, 38.86% NDF and 3.67% ASH (DM basis).

^2^ Contained 86.73% DM, 1.73% CP, 0.07% EE, 0.38% NDF and 0.241% ASH (DM basis).

^3^Contained 88.4% DM, 1.33% CP, 0.33% EE, 1.85% NDF and 0.41% ASH (DM basis).

Abbreviation: NDF_a_, apparent neutral detergent fiber; EE, ether extract; CP, crude protein; ASH, ashed sample weight.

^4^Estimated valued using NE_m_ values from the Nutrient Requirement of Small Ruminants (NRC 2007).

Ewes were weighed and blood sampled before the infusions on days 1, 9, 12, 16, 19, 23, 26, and 30 to measure plasma glucose, NEFA, and progesterone concentrations; plasma ghrelin concentration were analyzed from the day 30 samples. Transrectal ultrasounds (E. I. Medical imaging Ibex Pro–unit E. I. Medical L62L transducer) were carried out to evaluate ovarian activity on days 9, 16 and 26. During blood sampling, 10mL was collected from a jugular vein and transferred to tubes with disodium EDTA and benzamidine hydrochloride (1.6 and 4.7 mg/mL of blood, respectively) and placed on ice. After centrifugation for 25 min (1,800 ×*g*at 4°C), plasma was aliquoted and stored at -80°C until analysis.

### Animals, treatments, and sampling for Experiment II

The Experiment II was carried out to analyze hypothalamus mRNA concentration for neuropeptides and metabolic hormone receptors, ovarian mRNA stereological enzymes, hormones, and receptors, and to evaluate ovarian macroscopic structures, such as corpus luteum (CL) and preovulatory follicles.

The procedure of this Experiment was explained in Carranza Martin et al. [24]. Briefly, eighteen ewes (85.5 ± 1.06 kg and 2.5–3 in a five scale of body condition score; 21) had their estrus cycle synchronized and were assigned to the same treatments as mentioned in Experiment I. Ewes were infused every day before feeding for 13 days. Sheep in CO group were fed 1.5 times greater than the dietary maintenance requirements for a ewe based on the NRC requirement [25]. Ewes were given a total of 3.5 kg of dry matter (DM) intake/day, with 3.04 kg of corn silage, 0.32 kg of soy hull and 0.14 kg of soybean meal on DM basis. Ewes allocated to the FR and GA groups were given the same diet but at a rate of 80% of the NRC maintenance requirement [25] with a total feed intake of 1.87 kg/animal/day. As in Experiment I there were no feed refusals during the experiment. The same diet as described in Table 1 was given to the ewes in Experiment II.

Ewes were weighed and blood sampled before the infusion on days 1, 4, 9 and 13 to measure plasma glucose, NEFA, and progesterone concentrations. Blood samples were collected, aliquoted and stored as reported in Experiment I. On day 13 all ewes were euthanized, and the hypothalamus and ovaries were removed. The hypothalamus collection technique was as described by Glass et al. [26]. Ovaries were macroscopically evaluated within 5 minutes of harvest and then frozen. The left ovary and the hypothalamus of each ewe was then flash frozen in liquid N_2_ and stored at -80°C until analysis.

### Laboratory analysis for Experiments I and II

Plasma glucose and NEFA concentrations were measured using a colorimetric assay (1070 Glucose Trinder, Stanbio Laboratory,1261 North Main Street Boerne, Texas 78006; 96-well serum/plasma fatty acid kit non-esterified fatty acids detection 500 point kit, Zenbio Laboratory, NC; respectively) according to the manufacturer protocol. Plasma progesterone concentration was measured using commercial ELISA kit (BioMetallics In., Princeton Junction, NJ08550). The sensitivity of the assay was 0.4 ng/mL and the intra and inter assay coefficient of variation were 1.9% and 1%, respectively. The area under the curve (AUC) was calculated in Experiment I to measure the total of progesterone in one cycle (from d 9 to 30).

Plasma ghrelin was measured by RIA using an octanolyated ghrelin kit (Active Ghrelin Kit GHRA-88HK, Linco Research, St. Charles, MO), as described and validated for sheep previously [4]. The intra assay coefficient of variation was 7.8%.

Hypothalamus and ovaries were used to evaluate mRNA relative abundance. The genes evaluated were chosen due to their role in metabolism and reproduction functions. Genes analyzed from hypothalamus (Table 2) were *neuropeptide Y* (NPY), *NPY receptor 1* (NPY1R), *NPY receptor 2* (NPY2R), *agouti related protein* (AgRP), *pro-opiomelanocortin* (POMC), *cocaine and amphetamine regulated transcript* (CART), *melanocortin receptor 3* (MCR3), *melanocortin receptor 4* (MCR4), *kisspeptin* (Kiss1), *Kiss1 receptor* (Kiss1R), *gonadotrophin release hormone* (GnRH), *estrogen receptor 1* (ER1), *estrogen receptor 2* (ER2), *growth hormone release hormone* (GhRH), and *growth hormone receptor* (GH-R). Genes analyzed from ovaries were (Table 2): *aromatase* (CYP19), *steroid acute regulatory protein* (STAR), *ghrelin receptor* (GHSR), and *preproghrelin* (GHRL). The RNA was extracted by Trizol ® following the instructions of the manufacturer, and a sample of 175 ng of RNA was used to measure mRNA relative abundance using NanoString technology [27]. The mRNA relative abundance for each gene was normalized using 4 housekeeping genes (*GAPDH*, *ciclophilin A*, *PGK1*, *beta 2 microglobulin*, *and beta- actin*) using nSolver software.

**Table 2:** Hypothalamic and ovarian genes for the NanoString procedure.

Gene Name^1^	Accession Number
**Hypothalamus**	
Kiss1	NM_001306104.1
Kiss1R	NM_001318077.1
GnRH	U02517.1
ER1	AY033393
ER2	NM_001009737.1
NPY	NM_001009452.1
NPY1R	U62122.1
NPY2R	XM_012150937.2
AgRP	XM_015100491.1
POMC	NM_001009266.1
CART	XM_012145914.2
MCR3	XM_012108878.2
MCR4	NM_001126370.2
GHRH	XM_015091141.1
GH-R	NM_001009323
**Ovary**	
CYP19	AJ012153.1
STAR	NM_001009243.1
GHSR	NM_001009760.1
GHRL	DQ294307.1
**House keeping**	
Beta-actin	NM_001009284.2
Beta-2 microglobulin	NM_001308578.1
Ciclophilin A	NM_001190390.1
GAPDH	NM_001142516.1
PGK1	NM_001142516.1

^1^Kiss1, kisspeptin; Kiss1R, Kiss1 receptor; GnRH, gonadotrophin release hormone; ER1, estrogen receptor 1; ER2, estrogen receptor 2; GhRH, growth hormone release hormone; NPY, neuropeptide Y; NPY1R, NPY receptor 1; NPY2R, NPY receptor 2; AgRP, agouti related protein; POMC, pro-opiomelanocortin; CART, cocaine and amphetamine regulated transcript; MCR3, melanocortin receptor 3; MCR4, melanocortin receptor 4; GhRH, growth hormone release hormone; GH-R, growth hormone receptor; CYP19, aromatase; STAR, steroidogenic acute regulatory protein; GHSR, Ghrelin receptor; GHRL, preproghrelin; GADPH, glyceraldehyde 3-phospate dehydrogenase; PGK1, Phosphoglycerate kinase 1.

The weight (g) of the right and left ovary from each ewe was recorded and a caliper was used to measure the height, width and length (cm).The number of CL and preovulatory follicles (>6mm) were counted. The volume (cm^3^) of both ovaries per ewe was calculated as described by Chen et al. [28].

### Statistical analysis for Experiments I and II

Data was analyzed with a mixed procedure of SAS (9.4) as a randomized design. Plasma glucose, NEFA, plasma progesterone concentrations, and BW were analyzed as repeated measuresin time using the MIXED procedure of SAS (9.4, SAS Institute, Cary, NC). The model included the fix effect of treatment, time, their interaction, and the random effect of pen. Plasma ghrelin concentration and mRNA relative abundance were analyzed by PROX MIXED model (SAS Institute, Cary, NC), using a similar model but without time and its interaction. For mean separation on the time by treatment interaction the SLICE option of SAS was used. If there was no time by treatment interaction, a non-orthogonal contrast was used as a mean separator, comparing the three treatments among each other. Significance for main effects was set at P ≤ 0.05 and tendencies were determined at P ≤ 0.10 and P > 0.05.

## Results

### Experiment I

**Fig 1:**
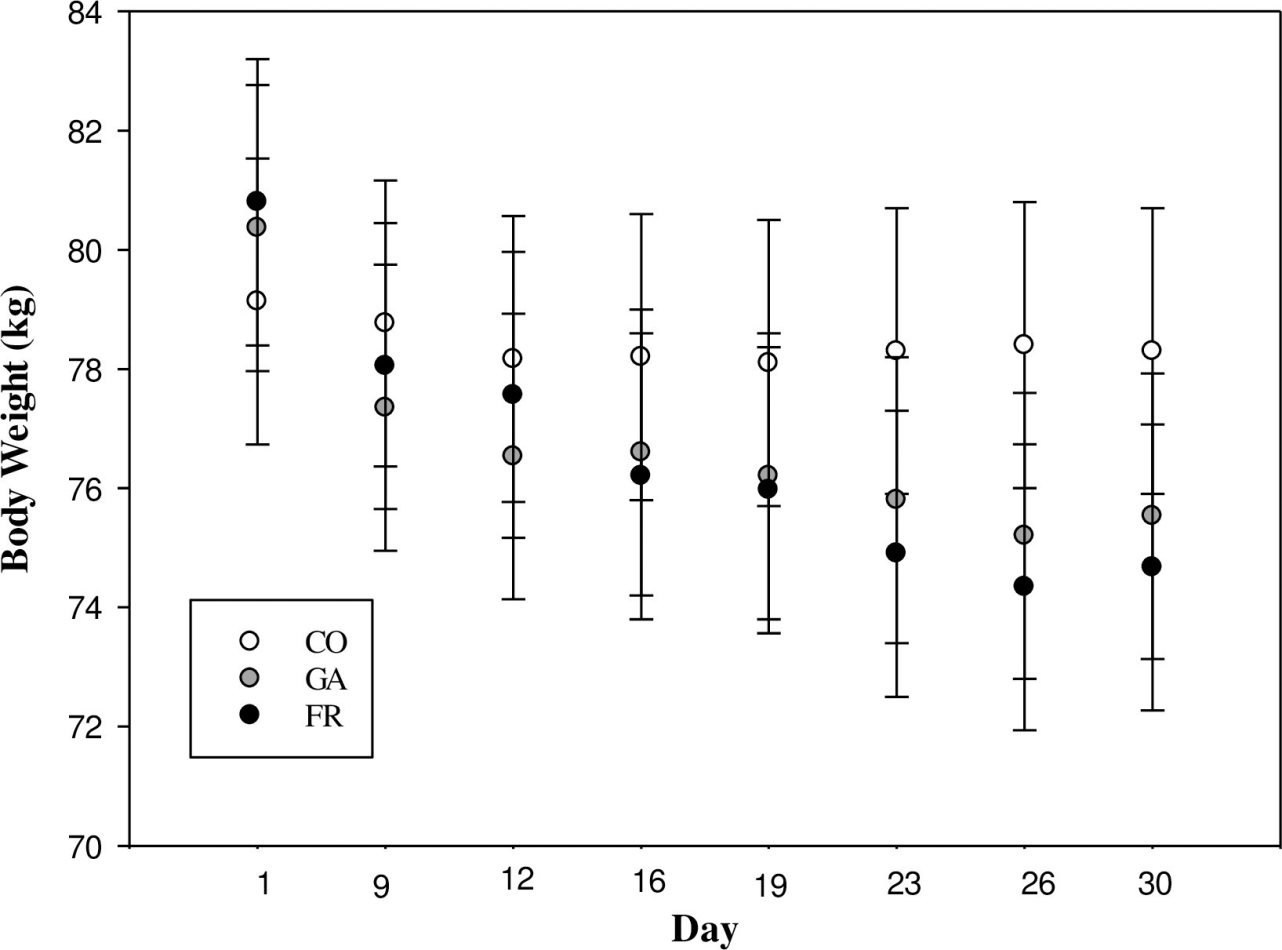
Body weight (kg) during 30 days of treatment of synchronized ewes. Treatments were: (i) Control (CO): ewes fed a diet that provided the maintenance nutrient requirements for a ewe and given a daily subcutaneous (SC) saline infusion (0.1mL/kg body weight, BW); (ii) Feed restriction (FR): ewes were fed a diet restricted to 80% of maintenance and given a daily SC saline infusion (0.1mL/kg BW); (iii) Ghrelin antagonist (GA): the same restricted diet as the FR and given a daily SC ghrelin antagonist infusion (7.5μg/Kg BW of [D-Lys3]-GHRP-6 diluted into 0.1ml/kg BW of saline solution). The FR and GA treatment decrease the BW in comparison with CO showing a time by treatment interaction (P = 0.06).

**Fig 2:**
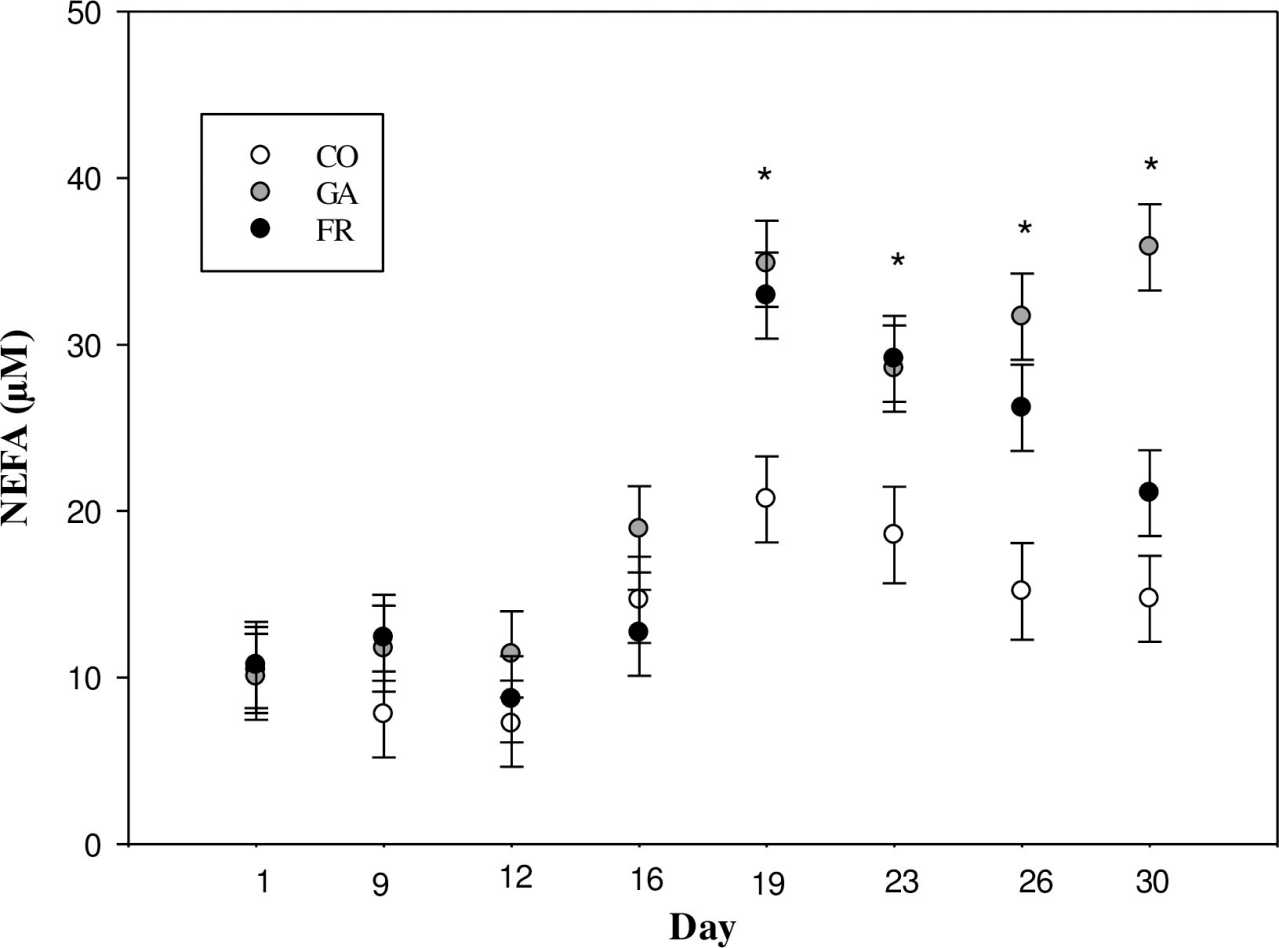
Plasma NEFA (μM) concentration during 30 days of treatment of synchronized ewes. Treatments were: (i) Control (CO): ewes fed a diet that provided the maintenance nutrient requirements for a ewe and given a daily subcutaneous (SC) saline infusion (0.1mL/kg body weight, BW); (ii) Feed restriction (FR): ewes were fed a diet restricted to 80% of maintenance and given a daily SC saline infusion (0.1mL/kg BW); (iii) Ghrelin antagonist (GA): the same restricted diet as the FR and given a daily SC ghrelin antagonist infusion (7.5μg/Kg BW of [D-Lys3]-GHRP-6 diluted into 0.1ml/kg BW of saline solution). The FR and GA ewes increased their NEFA plasma concentration during the experiment in comparison with CO showing a time by treatment interaction (P = 0.01). * indicate treatments differences (P ≤ 0.04) evaluated using the SLICE option in SAS.

Ewes allocated to the FR and GA treatments had similar plasma ghrelin concentrations (P = 0.60; Table 3). The FR and GA treatments however, showed greater plasma ghrelin concentrations than the CO group (P<0.01). There tended to be a treatment by time interaction in BW between CO and, FR and GA (P = 0.06). Ewes in the FR and GA treatment groups showed a trend to decrease BW during the experiment in comparison with the CO ewes (BW (kg): CO initial 79.13 ± 3.01 to final 78.3 ± 2.63; GA initial 80.37 ± 3.01 to final 75.53 ± 2.7; FR initial 80.8 ± 2.69 to final 74.67 ±1.87; Fig 1). There were no differences in plasma glucose concentration among groups (P≥0.25; Table 3). There was a treatment by time interaction for plasma NEFA concentration (P = 0.01). Feed restricted groups increased their plasma NEFA concentrations over the course of the experiment whereas CO NEFA plasma concentration did not change (NEFA (μM): CO initial 10.44 ± 1.74 to final 14.72 ± 3.21; GA initial 10.04 ± 1.39 to final 35.85 ± 3.7; FR initial 10.75 ± 2.34 to final 21.08 ± 2.34; Fig 2).

**Table 3:** Experiment I: Plasma ghrelin, glucose, and progesterone concentrations in ewes with a maintenance diet (CO), restricted diet (FR) and restricted diet with ghrelin antagonist infusion (GA) for 30 days.

	Treatments			SEM	P- Values			
	CO	GA	FR		CO vs GA	CO vs FR	GA vs FR	Treat by Time
Ghrelin (pmpl/mL)	16.11	29.13	31.46	3.51	<0.01	<0.01	0.60	
Glucose (mg/dl)	55.43	54.48	54.54	1.62	0.70	0.72	0.98	0.25
Progesterone (ng/ml)	4.96	5.77	5.37	0.26	0.03	0.27	0.29	0.20
Prog AUC^1^	85.57	102.4	95.66	7.58	0.17	0.40	0.54	

^1^ AUC: Area Under the Curve. Means were calculated based on the overall values of each treatment.

All ewes presented an active estrus cycle. The active estrus cycle was characterized by follicle growth, CL structure, and an increase in plasma progesterone concentration on 17 ± 0.6 days. Plasma progesterone concentration of the CO treated ewes was lesser than the GA treated ewes (P < 0.05), but plasma progesterone for the CO and GA treatments were not different to the FR treated ewes (P ≥ 0.20). There was no difference among treatments for plasma progesterone concentration as quantified by the AUC (P ≥ 0.17; Table 3).

### Experiment II

**Fig 3:**
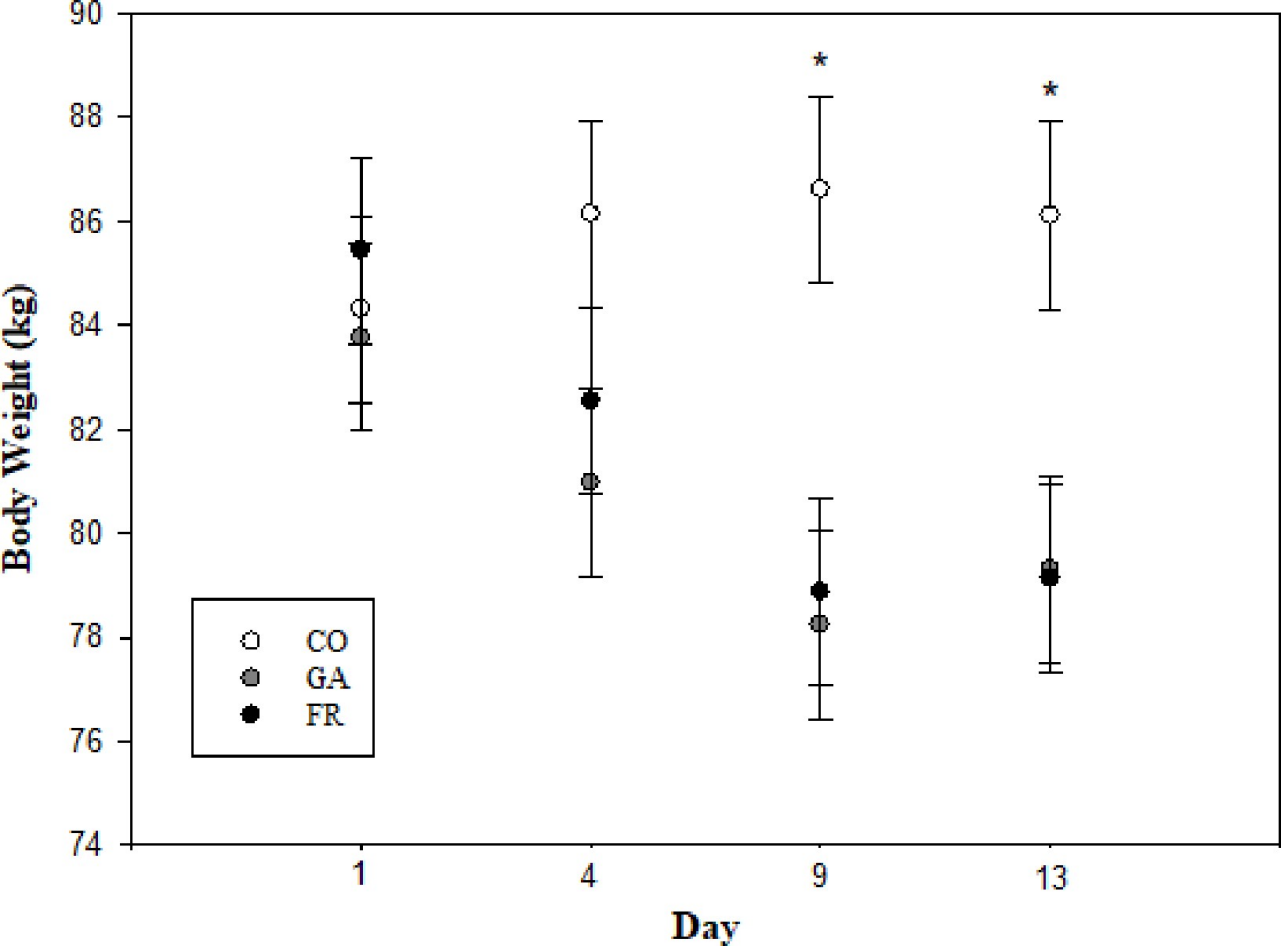
Body weight (BW, kg) during 13 days of treatment of synchronized ewes. Treatments were: (i) Control (CO): fed 1.5 times greater than the dietary maintenance and given a daily subcutaneous (SC) saline infusion (0.1mL/kg BW) (ii) Feed restriction (FR): ewes were fed a diet restricted to 80% of maintenance and given a daily SC saline infusion (0.1mL/kg BW); (iii) Ghrelin antagonist (GA): the same restricted diet as the FR and given a daily SC ghrelin antagonist infusion (7.5μg/Kg BW of [D-Lys3]-GHRP-6 diluted into 0.1ml/kg BW of saline solution). Time by treatment interaction P-value < 0.01. * indicate treatments differences (P ≤ 0.05) evaluated using the SLICE option in SAS.

There was a treatment by time interaction in BW (P< 0.01). Ewes allocated to the FR and GA groups decreased their body weight over the course of the experiment compared with the body weight of ewes allocated to the CO group that did not change throughout the experiment (BW (kg): CO initial 85.28 ± 3.55 to final 86.1 ± 3.36; GA initial 83.77 ± 3.55 to final 79.17 ± 3.23; FR initial 85.43 ± 3.64 to final 79.13 ± 3.08; Fig 3).

Plasma progesterone concentration was not different among the treatments (P ≥ 0.21). Ovaries from ewes allocated to the GA treatment had less volume (P = 0.02) and tend to weighed less (P = 0.07) than ovaries from the CO treated ewes. There were no differences in the volume and weight of ovaries from the GA and FR treated ewes (P ≥ 0.34). Likewise, there was no difference in ovary volume or weight between CO and FR treatments (P ≥ 0.14; Table 4). There was no difference in the number of CL and preovulatory follicles among the treatment groups (P ≥ 0.40).

**Table 4:** Experiment IImean (± SEM) body weight, plasma progesterone concentration, ovarian volume, weight, number of corpus luteum and number of follicles in ewes treated with a maintenance diet (CO), restricted diet (FR) and restricted diet with ghrelin antagonist infusion (GA) for 13 days.

	Treatments			SEM	P-Values			
	CO	GA	FR		CO vs GA	CO vs FR	FR vs GA	Treat by Time
Progesterone (ng/ml)	3.58	4.40	4.61	0.5	0.21	0.70	0.40	0.50
**Ovaries variables**^**1**^								
Ov. Vol (cm^3^)	2.3	1.67	1.92	0.18	0.02	0.15	0.34	
Ov. W (g)	2.55	1.9	2.04	0.21	0.07	0.14	0.65	
CL	1.5	1.58	1.25	0.28	0.83	0.53	0.40	
Follicle	0.67	1.17	1.08	0.23	0.14	0.21	0.80	

^1^ Ov. Vol: Ovaries volume; Ov. W: Ovaries weight; CL: Corpus luteum per ovary; Follicle: Follicles bigger than 6mm per ovary. Means were calculated based on the overall values of each treatment.

The relative expression of mRNA for Kiss1 in the hypothalamus was not different among groups (P ≥ 0.94). The ewes in the FR treatment however, had greater mRNA relative expression of Kiss1R than CO (P < 0.01). The Kiss1R mRNA relative expression in GA ewes tended to be greater than CO (P = 0.10) and was not different from the FR treated ewes (P = 0.22) (Table 5). The relative expression of mRNA for GnRH, ER1 and ER2 was not different among groups (P ≥ 0.13) (Table 5). The NPY mRNA relative expression (Table 5) was greater (P = 0.03) in the FR treated ewes compared with the CO treated ewes. The FR treated ewes tended to have greater mRNA relative expression for NPY (P = 0.06) when compared with the GA treated ewes; however, there were no differences in NPY1R and NPY2R mRNA relative expression (P≥0.36) among groups (Table 5). The mRNA relative expression for AgRP was not different among groups (P>0.10; Table 5). Neither were there differences (P ≥ 0.12) between groups for the anorexigenic neuropeptides, POMC and CART, nor the receptors MCR3 and MCR4 mRNA relative expression (Table 5). The GhRH mRNA relative expression was greater in GA treated ewes compared with the ewes in the CO group (P = 0.04). There was a trend for a greater relative expression of GhRH mRNA from the FR treated ewes compared with the ewes from the CO treatment (P = 0.07; Table 5). There were no differences in GH-R mRNA relative expression between groups (P ≥ 0.15) (Table 5). There were no differences in ovarian mRNA relative expression of CYP19, STAR, GHS-R, and GHRL among treatments (P ≥ 0.11) (Table 5).

**Table 5:** Hypothalamic and ovary mRNA relative expression of receptors, growth hormone release hormone and steroidogenic enzymes in ewes with a maintenance diet (CO), restricted diet (FR) and restricted diet with ghrelin antagonist infusion (GA) for 13 days.

	Treatments			SEM	P values		
	CO	GA	FR		CO vs GA	CO vs FR	FR vs GA
**Hypothalamus genes**							
KISS1	302.38	301.55	304.19	24.29	0.98	0.96	0.94
KISS1-R	10.99	13.96	16.12	1.20	0.10	<0.01	0.22
GnRH	33.41	41.38	56.98	10.45	0.60	0.13	0.31
ER1	273.30	266.35	287.31	11.38	0.68	0.42	0.24
ER2	49.42	42.06	50.54	4.35	0.25	0.86	0.19
NPY	358.64	378.89	529.37	51.29	0.78	0.03	0.06
NPY1R	213.93	212.63	215.10	10.05	0.93	0.93	0.86
NPY2R	79.35	78.41	84.78	4.54	0.89	0.43	0.36
AgRP	28.99	37.87	74.38	17.77	0.74	0.12	0.20
POMC	460.48	509.54	509.29	52.33	0.53	0.53	0.99
CART	800.58	648.90	773.42	81.73	0.21	0.82	0.30
MCR3	31.07	25.14	33.85	3.19	0.23	0.56	0.10
MCR4	11.05	10.52	11.18	1.72	0.83	0.96	0.79
GhRH	76.99	119.51	114.16	13.45	0.04	0.07	0.80
GH-R	118.34	128.84	132.81	6.74	0.29	0.15	0.68
**Ovary genes**							
CYP19	5.72	28.92	3.61	9.58	0.13	0.88	0.11
Star	137.7	106.3	61.8	64.04	0.64	0.43	0.74
GHS-R	1.74	3.85	3.68	0.97	0.17	0.20	0.90
GHRL	1.4	2.88	2.11	0.8	0.26	0.57	0.66

^1^Kiss1, kisspeptin; Kiss1R, Kiss1 receptor; GnRH, gonadotrophin release hormone; ER1, estrogen receptor 1; ER2, estrogen receptor 2; NPY, neuropeptide Y; NPY1R, NPY receptor 1; NPY2R, NPY receptor 2; AgRP, agouti related protein; POMC, pro-opiomelanocortin; CART, cocaine and amphetamine regulated transcript; MCR3, melanocortin receptor 3; MCR4, melanocortin receptor 4; GhRH, growth hormone release hormone; GH-R, growth hormone receptor; CYP19, aromatase; STAR, steroidogenic acute regulatory protein; GHS-R, ghrelin receptor; GHRL, preproghrelin.

## Discussion

Negative energy balance impacts reproduction by affecting the estrus cycle, pregnancy success and/or the number of offspring in sheep [29]. A suggested link between this nutritional status and reproduction is the hormone ghrelin, due to its association with the hypothalamus-pituitary-gonadal axis [30].

In the current experiment a small feed restriction did not change plasma progesterone concentration and follicular activity, despite the changes in plasma NEFA and ghrelin concentrations. One explanation is that the restriction of 20% was not low enough to provoke an impact on reproduction. Another explanation could be that ewes, previous to this study, were well fed to maintain a good BCS. For that reason, ewes had energy reserves, such as adipose tissue, that allowed them to continue with the reproductive cycle despite having a feed restriction. The endocrine responses to under nutrition in ewes are dependent on the initial body energy reserves of the animal [31].

Plasma ghrelin concentration has been reported to increase during fasting and the pre-prandial period [2]. Plasma ghrelin concentration was greater for ewes on the feed restricted diets (FR and GA groups; Experiment I). In addition, as discussed in Carranza Martin et al. [24], ghrelin concentration in a short period of time showed a tendency to be greater in GA ewes in comparison with CO ewes (26.43 pM/mL vs. 14.68 pM/mL, respectively)but there were no differences in ghrelin concentrations between theCO and FR (26.16 pM/mL).

The GA and FR ewes decreased BW in comparison with CO ewes during both Experiments, I and II. Also, ewes from FR and GA treatments in Experiment I increased their plasma NEFA concentration. In ruminants, NEB is associated with a loss in BW and increased plasma NEFAconcentration. These results showed that in both experiments ewes from FR and GA were in a NEB.

In our hypothesis we expected that FR ewes would suffer an estrus cycle disruption due to ghrelin’s action on the ovary, which would be reflected in a low plasma progesterone concentration. Furthermore, it was expected that the infusion of a ghrelin antagonist would overcome the estrus cycle disruption. Our study demonstrated no difference between FR and CO ewes for plasma progesterone concentration in both experiments. This contrasts with a study in rats that used the intra-ventricular infusion of 3 nM of ghrelin to cause a decrease in serum progesterone concentration [32]. The supraphysiological doses of ghrelin used in this study, however, may have contributed to the decrease in plasma progesterone concentration in the rats [32]. Moreover, others have reported that ghrelin concentration increased and LH concentration decreased when feed restricting cows and heifers but blood progesterone concentration did not change [33]. The restricted period implemented by Chouzouris et al. [33] was shorter than the period used in Experiment I; but it had a similar time period as Experiment II. It is possible that in the bovine and ovine, blood progesterone concentration is not associated with plasma ghrelin concentration. However, in the current study, administration of ghrelin antagonist for a period of 30 days increased plasma progesterone concentrations. We cannot explain however, the physiological mechanism behind the increase in plasma progesterone concentration when a ghrelin antagonist is given to a ewe.

Based on our hypothesis we were expecting to find a lesser volume in the ovaries of the FR ewes compared with the ovaries in the CO ewes because of the lack of large structures such as corpus luteum and follicles within the ovary. In addition, we expected a lesser mRNA abundance of the steroidogenic enzymes in the ovaries of the FR ewes than the CO ovaries. These effects would be counteracted with the use of the ghrelin antagonist. From our results, blocking the receptor with a ghrelin antagonist did not change the relative mRNA expression within the ewes’ ovarian tissue. The weights and volume of the CO ovaries however, were greater when compared with the ovaries of the GA treated ewes. The number of CL and follicles were not different for either of the two treatments; which leads us to assume that the increase in the weight and volume could be due to a greater amount of connective tissue or smaller structures, such as primary and secondary follicles. The presence of GHS-R has been reported in the stroma, the ovarian surface epithelium, primary and secondary follicles, granulosa cells, and CL in ewes [34]. Chronic ghrelin infusion decreased the diameter of follicles and CL in rats and decreased the diameter of other ovarian structures and, as a consequence, the whole ovarian volume was smaller in the treated animals compared with the control group [35]. Despite the difference observed in the ghrelin antagonist and control group, ewes in feed restriction alone did not present differences compared with the other treatments.

The addition of ghrelin (10 and 100 ng/mL) in bubaline CL culture produce a decrease in cytochrome P45011A1and 3-beta-hydroxysteroid dehydrogenase mRNA expression, but did not show a decrease in STAR mRNA expression [36]. The addition of ghrelin to *in-vitro* oocyte during maturation demonstrated a decrease in aromatase enzyme protein in pigs [37]. The lack of modification in the relative expression of mRNA of CYP19, STAR, GHS-R, and GHRL, in the present study could be explained by the short-term restriction (13d) or that the change in plasma ghrelin concentration was not enough to show differences in Experiment II.

The binding of Kiss1 to its hypothalamic receptor (Kiss1-R) produces the secretion of the GnRH that regulates the secretion of LH and follicle-stimulating hormone (FSH) in rats [38]. Ghrelin administration (intravenous, 3nM/250μL) down regulates Kiss1mRNA expression without changing the Kiss1-R mRNA abundance [39]. The mRNA expression of hypothalamic Kiss1 and Kiss1-R decrease after short-term fasting with an attenuation of the hypothalamus-pituitary-gonadalaxis in monkeys (*Macaca mulatta*) [40]. In contrast, we found a greater Kiss1-R mRNA abundance in FR and GA ewes compared with the Kiss1-R mRNA abundance of CO ewes. There was no change in the abundance of Kiss1 and GnRH mRNA expression between groups. We therefore suggest that changes in the expression of mRNA for Kiss1-R are not associated with the mRNA expression of GnRH. The Kiss1 neurons express both estradiol receptors (ER; 1 and 2). Estradiol ER1 has been implicated in the positive feedback LH surge and ovulation [41] in mice. Additionally, GHS-R and ER1 are co-expressed in many hypothalamic areas in mice [42]. However, in the present study ER1 and ER2 mRNA expression were not different between treatments.

Another possibility for the effect of ghrelin on theactivity of the hypothalamus-pituitary-gonadal axis is through orexigenic neuropeptides. We hypothesized that FR treated ewes would present an increasein orexigenic neuropeptides and decrease anorexigenic peptides. These changes would affect the relative expression of mRNA of Kiss1 and/or GnRH, inducing anestrus in the ewe. Changes in plasma concentration of ghrelin because of feed restriction had been associated with changes in mRNA expression of the neuropeptides NPY, AgRP and POMC [4, 43]. In Experiment II we observed an increased in mRNA abundance for NPY associated with feed restriction. The mRNA abundance for NPYwas less when feed restricted ewes were infused with a ghrelin antagonist. Chronic administration of NPY causes reproductive failure [44]. The effect of NPY on GnRH varies depending on the physiological stage of the animal. In fed mice, acute infusion of NPY increases GnRH secretion. However, chronic administration of NPY causes reproductive failure [44] by inhibiting GnRH neurons and disrupting the hypothalamus-pituitary-gonadalaxis [8]. Besides the significant increase in the abundance of NPY mRNA in FR ewes, there was no change in GnRH mRNA expression. We did not found differences in the expression of the ER1, ER2, NPY1R, NPY2R, ARP, POMC, CART, MCR3, and MCR4 mRNA, we suggest that it is possible that the ewes energy reserves, and or the 13 days of food restriction was insufficient time to observe these changes.

We expected that the relative expression of mRNA GhRH in FR would be greater than the GA and CO groups. Ghrelin stimulates growth hormone (GH) secretion [5] and also has a synergistic effect with GhRH on the hypothalamus [45]. In our results we found that GhRH mRNA relative expression was greater in FR and GA groups, even when the ghrelin receptor was blocked by [D-Lys-3]-GHRP-6. The infusion of NEFA into rats increases plasma GH concentration [46]. It is possible that the secretion of GH could be regulated via changes on GhRH caused by the infusion of NEFA. Therefore,the increase in plasma NEFA concentration in the current experiment may explain the lack of change in relative mRNA expression of GhRHin the [D-Lys-3]-GHRP-6 infused ewes.

## Conclusion

We can conclude that a small feed restriction does not modify plasma progesterone concentration and ovarian cyclicity in ewes with a good body condition score. Short term ghrelin antagonist infusion in feed restricted ewes does not affect ovarian follicles and CL number, the mRNA expression of STAR and aromatase. However, ghrelin antagonist modifies ovarian volume and weight. A short term infusion of ghrelin antagonist changes the abundance of NPY and Kiss1-R mRNA in the hypothalamus of feed restricted ewes but not in Kiss1, GnRH, ER1, ER2, NPY1R, NPY2R, AgRP, POMC, CART, MCR3, and MCR4 mRNA abundance. The abundance of GhRH mRNA was increased in feed restricted ewes in both groups, FR and GA; however, the infusion of ghrelin antagonist did not have an effect on GH-R mRNA abundance.

## Supporting information

S1 Data (XLSX)
